# Effect of Ramipril on Cardiac Autonomic Neuropathy in Patients With Type II Diabetes Mellitus

**DOI:** 10.7759/cureus.36209

**Published:** 2023-03-15

**Authors:** Chaitali A Chindhalore, Ganesh N Dakhale, Prathamesh H Kamble, Bharatsing D Rathod, Sunita Kumbhalkar, Mrunal S Phatak

**Affiliations:** 1 Pharmacology, All India Institute of Medical Sciences, Nagpur, Nagpur, IND; 2 Physiology, All India Institute of Medical Sciences, Nagpur, Nagpur, IND; 3 General Medicine, All India Institute of Medical Sciences, Nagpur, Nagpur, IND

**Keywords:** ewing"s autonomic function testing, heart rate variability (hrv), ramipril, quinapril, ace (angiotensin converting enzyme), diabetic autonomic neuropathy

## Abstract

Background

Cardiovascular autonomic neuropathy (CAN), an important form of DAN is caused by the impairment of the autonomic nerve fibers that innervate the heart and blood vessels and leads to abnormalities in cardiovascular dynamics. The earliest finding of CAN, even at the subclinical stage, is a decrease in heart rate variability (HRV).

Objective

The objective is to assess the effect of ramipril 2.5mg once daily on cardiac autonomic neuropathy in type II DM patients as an add-on to a standard antidiabetic regimen for a duration of 12 months.

Materials and methods

A prospective, open-label, randomized, parallel-group study was conducted on type II DM with autonomic dysfunction. Patients in Group A received tablet ramipril 2.5mg daily along with the standard antidiabetic regimen which consist of Tab Metformin 500mg twice a day and Tab Vildagliptin 50mg twice a day and group B received only the standard antidiabetic regimen for 12 months.

Results

Among 26 patients with CAN, 18 patients completed the study. After one year in group A, Delta HR value increases from 9.77±1.71 to 21.44±8.44 and the E:I ratio (ratio of the longest R-R interval during expiration and shortest R-R interval during inspiration) improved from 1.23±0.35 to 1.29±0.23 signifying significant improvement in parasympathetic tone. Results of the postural test showed significant improvement in SBP. Analysis of HRV by time domain method showed that the standard deviation of RR (SDRR) interval and Standard deviation of differences between adjacent RR interval (SDSD) value increased significantly in group A. Analysis of HRV frequency domain indices showed that LFP:HFP ratio improved after treatment in ramipril group indicating improvement in sympatho-vagal balance.

Conclusion

Ramipril improves parasympathetic component more as compared to sympathetic component of DCAN in type II DM. Ramipril could be a promising option having favorable long-term outcomes in diabetic patients especially when treatment begins at subclinical stage.

## Introduction

Diabetic autonomic neuropathy (DAN) is a serious but unfortunately least recognized complication of diabetes despite its significant negative impact on survival and quality of life [1]. Cardiovascular autonomic neuropathy (CAN) is an important form of DAN that encompasses damage to the autonomic nerve fibers that innervate the heart and blood vessels resulting in abnormalities in heart rate control and cardiovascular dynamics [2].

CAN may be present at diagnosis with presenting symptoms like resting tachycardia, arrhythmias, intraoperative cardiovascular instability, asymptomatic myocardial ischemia, and infarction, and prevalence increases with age, duration of diabetes, obesity, smoking, and poor glycemic control. A study including 1171 patients with both types of diabetes mellitus reported impaired heart rate variability (HRV) tests in 25.3% of type 1 and 34.3% of type 2 patients [3].

Meta-analyses of 15 study results support an association between CAN and increased risk of mortality when autonomic dysfunction was defined as the presence of two or more abnormalities of tests for HRV. A stronger association was observed in subjects with other comorbid complications that contributed to their higher mortality risk [4]. The ACCORD trial study group concluded that CAN was associated with increased mortality in the type 2 diabetes cohort, whether patients received intensive or standard glycemia treatment in the ACCORD cohort [5].

Earlier study findings suggest early detection and treatment of CAN at the subclinical stage as cardiovascular denervation is reversible in the early stage. By the time clinical manifestations occur, CAN has often reached a late stage, making management more difficult [6]. Hence, patients should be screened for autonomic dysfunction immediately after the diagnosis of type 2 diabetes.

A study by Pop-Busui [7] explained that the first manifestation of diabetic CAN affects vagus nerve, which is responsible for nearly 75% of parasympathetic activity. Vagal impairment leads to parasympathetic derangement and the sympathetic tone becomes predominant. Unnoticed and untreated for several years, it further progresses to sympathetic function damage. The progressive damage of the autonomic balance causes additional symptoms, including intolerance to exercise, orthostatic hypotension, and a further reduction in HRV.

A decrease in HRV is the earliest indicator of CAN even at the subclinical stage. HRV is a beat-to-beat variation in heart rate (i.e., in R-R intervals) which occurs due to continuous changes in the sympathetic and parasympathetic outflow to the heart. It is non-invasive and objective in the evaluation of cardiac autonomic function and can be performed by recording electrocardiogram at rest. HRV analysis enables the independent measurement of the sympathetic and parasympathetic components of the autonomic nervous system [8].

In the 1980s, Ewing et al. discovered five simple tests of short-term R-R alterations to identify CAN in patients with diabetes which consist of deep breathing test, Valsalva maneuver, Isometric hand grip, cold pressor test and lying to standing test/head-up tilt test. An American Diabetes Association statement describes these validated cardiac autonomic reflex tests (CART) in detail and recommends their use in the diagnosis of CAN. HRV with deep breathing is the most commonly used autonomic function test and has a specificity of approximately 80% [9].

Pathogenesis oriented interventions may promote some degree of reversal of established CAN. Researchers explored different medication approach like α lipoic acid [10] and aldosterone inhibitors [11] to treat CAN whose results were not much promising. Recently the EMPA-REG outcome trial [12] in T2DM demonstrated improvement in sympathetic tone due to empagliflozin along with reduction in cardiovascular events. Contrary, Liraglutide, a GLP-1 receptor agonist had reduced HRV with decrease in parameters of parasympathetic activity (RMSSD and HF power) suggesting negative impact on sympatho-vagal balance [13]. An experimental study in streptozotocin-induced diabetic rats, Coppey et al. [14] studied effect of angiotensin-converting enzyme inhibitors (ACEIs) on endoneurial outflow and nerve conduction velocity and concluded that ACEI has a potential to attenuate diabetic vascular and neural dysfunction.

Of the ACE inhibitors studied, a study by Didangelos et al. [15] demonstrated that treatment with quinapril for two years improves parasympathetic function of DCAN as expressed with the indices of deep breathing test. On the contrary, study by Malik et al. [16] reported that trandolapril may improve peripheral neuropathy in normotensive patients with diabetes but vibration-perception threshold, autonomic function, the neuropathy symptom and deficit score showed no improvement after 12 months of treatment with trandolapril. In a study by Ebbehoj [17], addition of metoprolol to ongoing ramipril therapy shows improvement in autonomic dysfunction.

Results are conflicting from above mentioned studies. At the same time, effect of medication might not be homogenous in same class. Considering the sequalae of CAN, efforts to reverse it by various drug therapies is of paramount importance. Despite of extensive literature search, we could not find any study which evaluated efficacy of ACEI in reversing CAN in the Indian population. Hence, present study was planned to explore potential of ramipril in reversing CAN in type II DM.

## Materials and methods

A prospective, open-label, randomized, parallel-group study was conducted at a tertiary care teaching institute in central India from July 2020 to July 2022 after getting approval from the Institutional ethics committee (IEC/Pharmac/51/20, date: March 13, 2020). The study was registered prospectively on the clinical trial registry India (CTRI/202/04/024723).

Sample size

The sample size was calculated based on the change in E/I index as a primary variable from the previous study [15], with effect size 0.19 & SD 0.12, Sample size calculated was 9 in each group. Considering 20% dropouts, the calculated sample was 11 in each group. the sample size was calculated using PS Power and sample size calculation version 3.1.6.

Inclusion criteria

Patients suffering from type II diabetes mellitus for ≤ 5 years, with Hb1Ac ≤ 8.5 who were diagnosed as suffering from autonomic dysfunction on screening were included. Among eligible patients, those who were willing to give written informed consent were recruited.

Exclusion criteria

Patients suffering from hypertension and on antihypertensive medications were excluded from the study. Patients who were suffering from concurrent debilitating illnesses like chronic renal failure, malignancy, cirrhosis, etc., based on history and symptoms were excluded. Subjects on drugs that are known to influence cardiac autonomic function like beta blockers or patients having a history of allergy to study medication were not included in the study. Also, lactating and pregnant women on the basis of history given by patients were excluded.

Randomization

Patients suffering from type II DM were screened for autonomic dysfunction. Patients were undergone Cardiac Autonomic Function Testing which include analysis of HRV by time domain and frequency domain method and Ewing’s validated autonomic function test during the screening visit. Patients were diagnosed to be suffering from CAN if they have at least two abnormal values and these values were considered baseline values. Among them, those who were willing to participate in the study were enrolled after taking written informed consent. Patients were assigned randomly in a 1:1 ratio to Group A or Group B using a computer-generated simple random number table. Allocation to the group was concealed by using a sealed envelope until intervention had been assigned. All patient Demographic data and relevant medical history were noted. Other baseline investigations include Hb1Ac, fasting and postprandial blood glucose, serum creatinine, lipid profile, and serum potassium level which were repeated at the end of the study.

Patients in Group A received a tablet of ramipril 2.5mg daily along with the Standard antidiabetic regimen which consist of Tab Metformin 500mg twice a day and Tab Vildagliptin 50 mg twice a day and group B received only a standard antidiabetic regimen for 12 months. Antidiabetic regimens were decided by treating physicians who were not aware of the group allotment of the patient. Patients were asked to come for a follow-up visit every three months for blood glucose monitoring and to check for compliance. Those patients having uncontrolled blood glucose levels requiring change in antidiabetic regimen were excluded during follow-up visits. Patients were asked to bring empty blister packs of medication to check for compliance. Eighty percent of adherence to treatment was considered as compliant. All investigations including AFT and laboratory investigations were repeated after 12 months (at the end of the study).

Study procedure for cardiac autonomic function testing

Before the autonomic assessment, subjects were instructed to avoid food preceding two hours before the testing and no coffee, tea, nicotine, or alcohol 24 hours prior to the testing. Drugs known to affect cardiac autonomic functions like anticholinergics (including antidepressants, antihistamines, and over-the-counter cough and cold medications), 9-α-flurocorticone, diuretics, and sympathomimetic (α and β agonist) and parasympathomimetic agents should be stopped after consultation with the physician for two days prior to testing. The autonomic assessment was conducted by analyzing HRV and Ewing’s validated autonomic function testing.

Instrumentation

Heart rate, beat-to-beat BP, and respiratory movements were continuously monitored using the digital data acquisition system PowerLab 26T (AD Instruments Systems Pvt Ltd, Australia). Continuous ECG was recorded in Lead II with the help of shielded cables and disposable Ag-AgCl electrodes. Chest movements were acquired using the respiratory belt transducer system for recording chest expansion. The belt for recording chest movements was wrapped around the chest at the level of the fourth intercostal space. Beat-to-beat arterial blood pressure was measured using a continuous noninvasive blood pressure system (ADI/FMS NIBP system, Australia). BP sensor was placed at the middle finger of the left hand and height correction for BP measurement was done. NIBP measurements was checked with absolute BP measurements obtained by an automated sphygmomanometer (OMRON). The ECG signal, respiratory and BP waveform were sampled at 1,000Hz (ADI) and beat to beat values of heart rate, RR interval, systolic BP, diastolic BP, and mean BP were stored for off-line analysis. The analysis was done by LabChart software which automatically detect QRS complexes and discard artefacts/abnormal beats. The distances between consecutive R-waves were computed.

Experimental procedure

The subjects who arrived, after following all the instructions, were asked to relax in a supine position on a medical examination table. Following instrumentation, patients were advised 15 minutes of rest after which the resting HRV was performed as described below.

HRV Analysis

For analysis of HRV, ECG was recorded in the supine position for 5 min. The subject was instructed to close the eyes and to avoid talking, moving hands, legs and body, coughing and sleeping during test. R-R intervals from ECG signals was detected using LabChart 8. Analysis of time-domain measures was carried out and following parameters were noted: standard deviation of the R-R intervals (SDRR), root square of the mean of the sum of the squares of differences between adjacent RR intervals (RMSSD) and standard deviation of successive differences between adjacent RRs (SDSD). SDRR is an evaluation of both sympathetic and parasympathetic activity on HRV, whereas RMSSD and SDSD are primary indicator of parasympathetic activity as stated by Vinik et al. [18].

Diagnostic assessment as explained by Vinik and Ziegler [2] of Spectral analysis involves decomposing the series of sequential R-R intervals into a sum of sinusoidal functions of different amplitudes and frequencies by several possible mathematical approaches. The power spectrum reflects the amplitude of the heart rate fluctuations present at different oscillation frequencies. The power spectrum of HRV has been shown to consist of three major peaks: (1) very-low frequency component (below 0.04 Hz), Low Frequency (LF-0.040 to 0.15) Hz and High Frequency (HF-0.15 to 0.4) Hz component. The very-low-frequency heart rate fluctuations are thought to be mediated primarily by the sympathetic system, and the LF fluctuations are predominantly under sympathetic control with vagal modulation, whereas the HF fluctuations are under parasympathetic control. LF/HF ratio was calculated which indicates sympatho-vagal balance.

Ewing’s Validated Cardiovascular Autonomic Testing

It consists of battery of simple noninvasive tests based on HR and BP responses to certain physiological stimuli/maneuvers [19,20]. Ewing and Clarke described Lying to standing test (Fall in SBP) and Isometric exercise test (Rise in DBP) tests for sympathetic function assessment. Tests for parasympathetic function assessment included Lying to standing test (30:15 ratio), and Heart rate response to deep breathing (E:I ratio) whereas Valsalva ratio reflects functioning of both components.

1. Deep breathing test: Patients were instructed to perform deep and regular breathing at the rate of six breaths per minute. For six cycles per min, the inspiration was done for 5 sec and expiration for 5 seconds. Normal response is increase in heart rate with inspiration and decrease in heart rate with expiration. Calculation was done from the tracing of respiration and ECG.

Parameters: Delta HR - difference between the maximal and minimal heart rate during inspiration and expiration respectively, averaged for six cycles. E:I ratio - ratio of the longest R-R interval and shortest R-R interval averaged over six cycles.

2. Valsalva maneuver: Valsalva maneuver (Longest RR interval during phase IV/shortest RR interval during phase II) was done in sitting position. The patient blows into a mouthpiece attached to sphygmomanometer. The expiratory pressure was kept at 40 mmHg for 15 seconds. A small air leak in system was done to prevent the closure of glottis during the maneuver. At the end of 15 seconds the pressure was released.

3. Isometric exercise (Handgrip test): The subject was instructed to perform isometric exercise using handgrip dynamometer. After the instruction the subjects were asked to grip using maximum force with their dominant hands for a few seconds. The maximum value of the three readings is considered as their maximal voluntary contraction (MVC). A mark was made on the dynamometer at 30% of MVC of the subject. Instruction had been given to the patient to maintain the sustained grip on the dynamometer up to mark for 4 minutes. Rise in diastolic BP as calculated by difference between highest DBP during the test and baseline DBP.

4. Postural test (change of posture from lying to standing test): The test was conducted after 10 min of supine rest. Patient was told to attain the standing posture within 3 seconds and recordings was taken. The blood pressure and heart rate are recorded at baseline and serially at 0.5th, 1st, 2nd, 2.5th and 5th min. Fall in systolic BP and 30:15 ratio (ratio between the longest R-R interval at or around the 30th beat and the shortest R-R interval at or around the 15th beat) were calculated.

Ewing’s score: All test results will be defined as normal, borderline and abnormal as described by Ewing and Clark’s classification of autonomic test results. Then scores will be assigned as follows: 0 for normal, 1 for borderline and 2 for an abnormal value. The sum of all five scores will be defined as Ewing’s score. We will define definite parasympathetic dysfunction as a score of ≥ four and definite sympathetic dysfunction as a score ≥ two.

Outcome parameters

1. Change in heart rate (Delta HR) and E:I ratio during deep breathing test from baseline to the end of study

2. Change in VR during Valsalva maneuver test from baseline to the end of study

3. Rise in DBP after isometric exercise from baseline to the end of study

4. Fall in SBP and 30:15 ratio during postural test from baseline to the end of study

5. Change in SDRR, SDSD and RMSSD from baseline to end of the study

6. Change in LF/HF ratio from baseline to the end of study

Statistical analysis

Data were compiled in Microsoft excel sheet and expressed as mean±SD. Continuous variables (normal distribution) were compared between groups by unpaired t-test and within group by paired t-test. Value of P ≤ 0.05 was considered statistically significant. Graph pad prism 9 software was used for analysis.

 Ethical consideration

The study was conducted after getting approval from Institutional ethics committee. Before recruitment, written informed consent was taken from participants after explaining the nature of study. Participants were informed that their participation in the study was voluntary, and they have a right to quit from study any time. Also, they were informed that their identity and responses were kept confidential and were analyzed only as a part of cohort.

## Results

Sixty-five type II diabetic patients were screened for autonomic dysfunction. Of these, 26 patients were found eligible for enrollment. Total 23 patients were randomized out of which 11 patients were allocated to group A and 12 patients to group B. In group A, one patient declined intervention during follow up and one patient was not traceable. So, in group A, nine patients completed the study. In group B, during follow-up period, two patients developed hypertension and one patient declined to be a part of study. Hence, in group B also, data from nine patients were analyzed. Both groups had similar baseline characteristics (Table 1).

**Table 1:** Baseline demographic and clinical profile of patients in Group A and Group B. Unpaired “t” test

Sr No	Parameter	Group A (n= 9)	Group B (n=9)	P-value
1	Age (Years)	53.33±5.74	48.22±12.31	0.27
2	M:F ratio	2:1	2:1	
3	Duration of DM (Years)	3.11±1.53	2.55 ±1.23	0.41
4	Systolic blood pressur	116.9 ±5.2	113.3±4.79	0.15
5	Diastolic Blood pressure	77.33±3.07	78.22± 3.8	0.55
6	Hb1A1c (%)	7.44 ±0.73	7.22±0.38	0.43
7	Fasting blood glucose (mg/dL)	134±15.76	135.5±26.15	0.88
8	Postprandial blood glucose (mg/dL)	182.3± 47.21	178.8±27.66	0.84
9	Total cholesterol (mg/dL)	167.1±40.79	163.1±27.11	0.81
10	HDL (mg/dL)	41.07±8.51	40.1±5.84	0.78
11	LDL (mg/dL)	107.9±34.88	119.4±13.23	0.36
12	TG (mg/dL)	156.8±30.98	163.2±58.62	0.77
13	Creatinine (mg/dL)	0.83±0.12	0.81±0.03	0.8
14	Serum Potassium (mmol/L)	4.39± 0.41	4.28±0.49	0.62

After one year in group A, Delta HR value increases from 9.77±1.71 to 21.44±8.44 and E:I ratio improved from 1.23±0.35 to 1.29±0.23 which indicates significant improvement in parasympathetic tone. Results of postural test showed significant improvement in SBP (p=0.014). Significant improvements were observed in Ewing’s parasympathetic score (p=0.0002) and Ewing’s sympathetic score (p=0.01) Though, in group B, delta HR value increased from 9.33±3.04 to 12.33±3.35 (p=0.027) but improvement in delta HR value is significantly more in Gr A as compared to Gr B (p=0.021). Improvement in Ewing’s parasympathetic score was significantly more in Group A as compared to Gr B (p=0.04) (Table 2).

**Table 2:** Comparative analysis of cardiovascular autonomic reactivity (n=18). * p<0.05; **p<0.01; ***p<0.001; student’s “t” test; HR: Heart rate; SBP: systolic BP; DBP: Diastolic BP; E:I ratio-longest RR interval/shortest RR interval

Sr No		Group A (Ramipril+Std Antidiabetic drugs)		Group B (Std Antidiabetic drugs)	
		Baseline	End of study	Baseline	End of study
1	Deep breathing test				
	Change in HR	9.77±1.71	21.44±8.44**	9.33±3.04	12.33±3.35*
	E:I ratio	1.23±0.35	1.29±0.23**	1.22±0.2	1.26±0.15
2	Valsalva ratio	1.35±0.30	1.58±0.38*	1.48±0.73	1.55±0.5
3.	Isometric exercise				
	Rise in DBP	13.56± 8.66	14.89±6.43	12.89±4.54	12.89±3.29
4.	Postural test				
	Fall in SBP	16.33±6.51	9±3.7*	13.67±4.21	11.33±3.31
	30:15 Ratio	1.05±0.06	1.07±0.04	1.06±0.06	1.08±0.1
5	Overall Parasympathetic score	2.22±0.44	0.33±0.5***	2.11±0.78	1±0.7*
6	Overall Sympathetic score	1.88±1.26	0.66±1*	1.55±0.88	1±0.86

Analysis of HRV by time domain method showed that, SDRR and SDSD value increased significantly in group A which indicates improvement in parasympathetic tone.

Analysis of HRV frequency domain indices (high frequency power [HFP], reflecting vagal tone, low frequency power [LFP], reflecting both vagal and sympathetic [predominantly] modulation, and their ratio [LFP/HFP], indicative of sympatho-vagal balance) were assessed in patients of both groups at baseline and one year after therapy which showed that LFP:HFP ratio improved after treatment in ramipril group indicating improvement in sympatho-vagal balance.

**Table 3:** Dynamics of parameter of autonomic activity (HRV) (n=18). *p<0.05; **p<0.01; student’s “t” test SDRR: Standard deviation of RR interval; SDSD: Standard deviation of differences between adjacent RR interval; RMSSD: Root square of the mean of the sum of the squares of differences between adjacent RR interval; LF: Absolute power of low-frequency band; HF: Absolute power of high-frequency band

Sr No	Parameter	Group A		Group B	
		Before	End of study	Before	End of study
1	Time domain				
	SDRR	25.08±11.1	30.41±12.43**	26.26±12.32	29.33±19.81
	SDSD	16.54±6.74	23.75±12.37*	19.80±9.67	26.35±19.37
	RMSSD	16.52±6.74	23.82±12.3*	19.95±10.75	26.19±19.5
2	Frequency domain				
	TP	732±594.8	941.8±724.2	781.6±801.1	975±1277
	LFP	240.5±236.6	233.3±227	216.3±107.6	286.5±474
	HFP	133.9±110.8	371±469.6	275±224.8	350±412
	LFP: HFP	1.72±0.71	0.95±0.63	1.3±0.99	0.97±0.48

## Discussion

CAN is an important predictor of cardiovascular outcome and mortality in T2DM. The present study assessed, the effect of ramipril on CAN in terms of improvement in parasympathetic and sympathetic tone through a battery of tests for the first time in the Indian population. The present study included only well-characterized, highly selected normotensive T2DM patients diagnosed to be suffered from CAN. Patients had similar baseline characteristics.

Results of our study reflect that ramipril significantly improved HRV as expressed with indices of deep breathing like delta HR and E:I ratio which suggests improvement in parasympathetic tone. Variation in HR during deep breathing is primarily mediated by the vagal innervation of the heart. The first manifestation of diabetic CAN tends to be related to vagus nerve damage, which is responsible for nearly 75% of parasympathetic activity. So, maybe, they are the first to improve with appropriate treatment. A study by Didangelos et al. observed improvement in DCAN and left ventricular dysfunction after 1 year of treatment with quinapril [21].

Considering neurovascular insufficiency as one of the etiologies, it was postulated that ACE inhibitors had a beneficial effect in reversing CAN through increases in nerve blood flow by promoting vasodilation. ACEIs act by preventing the generation of angiotensin II and also inhibit the breakdown of bradykinin. Angiotensin II, in addition to its role as a vasoconstrictor, stimulates aldosterone release. Aldosterone affects the autonomic nervous system with sympathetic activation and parasympathetic inhibition and causes impairment of the baroreflex response. Thus, effective ACE inhibition impacts very positively on cardiovascular outcomes.

Surprisingly, we observed improvement in delta HR in the control group also but to less extent when compared with ramipril. Like other diabetic complications, blood glucose optimization through strict glycemic control has a favorable effect on CAN. Previous study findings from the Steno-2 trial also stated that intensive multifactorial treatment (e.g., targeting hyperglycemia, hypertension, and dyslipidemia, including acetylsalicylic acid for secondary prevention) and targeted strict glycemic control reduced the incidence of autonomic dysfunction in type 2 diabetes [22].

The present study shows significant improvement in indices of postural test in terms of fall in SBP in the ramipril group. The change in posture from lying to standing puts hydrostatic stress on the venous return. The venous decrease due to the pooling of blood in the lower limbs results in a decrease in blood pressure which stimulates baroreflex resulting in the rise of the HR. The rise in HR raises the BP toward resting values. Thus, the postural test involves Baroreceptor and cranial nerves IX and X as afferents and sympathetic (adrenergic) and Parasympathetic (cardiovagal, cholinergic) as efferent fibers. Thus, the study findings of the postural test suggest significant improvement in the parasympathetic component and sympathetic component to some extent since there was no improvement in other indices for sympathetic function.

Current study findings show improvement in the Valsalva ratio after treatment with ramipril, but it was not significant. Valsalva maneuver is a more complex test; it encompasses a complex reflex arc involving both sympathetic and vagal pathways to the heart, sympathetic pathways to the vascular tree, and baroreceptors in the chest and lungs [11]. Thus, it is reasonable that the changes in Valsalva Index probably require longer time than the follow-up period of our study.

Current study findings showed significant improvement in overall Ewing’s parasympathetic and Ewing’s sympathetic score but improvement in parasympathetic component is more as compared to sympathetic component. Sympathetic improvement needs further treatment for extended duration which is a limiting factor of our study. Analysis of HRV by time domain method which quantify the variation in RR intervals showed significant improvement in SDRR, SDSD, and RMSSD, which suggest significant improvement in HRV from baseline after treatment with ramipril.

Frequency component of HRV was analyzed using fast Fourier transform (FFT) method. The power spectrum is divided into three frequency bands as VLF, LFP and HFP. In present study, though there were improvement in LFP and HFP indices, but it was not statistically significant. Present study reported significant reduction in LFP:HFP ratio which shows improvement in sympatho-vagal balance as LFP band is mediated by sympathetic and HFP band is under parasympathetic influence. A study by Kontopoulos et al. also reported reduction in LFP/HFP ratio with quinapril as compared to placebo [23].

Our study assessed effect of ramipril on CAN in type 2 DM for the first time in Indian population; however, limited sample size, comparatively short duration of study to assess change in autonomic function and chances of observer bias were major limitations. Repeated AFT to observe effect of ramipril on CAN at frequent interval might be more meaningful which was not feasible in present study due to limited resources. Though both the groups had similar baseline characteristics but during follow up, there might be difference in glycemic control which may also be a confounding factor.

## Conclusions

Diabetic CAN, a common but neglected disease-related complication is associated with poor prognostic outcomes. It may be attributable to functional or structural damage to the autonomic nervous system. Testing autonomic activity and reactivity using a simple test like change in HRV and BP in response to breathing, Valsalva maneuver, postural changes, and isometric exercise are helpful in diagnosing functional abnormalities and imbalance between the sympathetic and parasympathetic nervous system. Present study findings suggested that ramipril improved DCAN to a significant extent in type 2 diabetics. Ramipril improves the parasympathetic component more as compared to the sympathetic component. Improved autonomic balance is clinically important in predicting long-term outcomes in diabetic patients. ACE inhibition and especially ramipril could be a promising option for the treatment of CAN especially when treatment begins at the subclinical stage.

Since cardiac autonomic dysfunction is an important contributor to cardiovascular events like silent MI, malignant arrhythmias and sudden cardiac death, improvement of indices related to autonomic function in patients with DCAN may have a prognostic benefit.
